# Enhanced Osseointegration of a Modified Titanium Implant with Bound Phospho-Threonine: A Preliminary In Vivo Study

**DOI:** 10.3390/jfb8020016

**Published:** 2017-05-25

**Authors:** Yohei Okazaki, Kazuya Doi, Yoshifumi Oki, Reiko Kobatake, Yasuhiko Abe, Kazuhiro Tsuga

**Affiliations:** Department of Advanced Prosthodontics, Hiroshima University Graduate School of Biomedical and Health Sciences, 1-2-3, Kasumi, Minami-ku, Hiroshima 734-8553, Japan; okazaki-yoh@hiroshima-u.ac.jp (Y.Oka.); yos-oki14@hiroshima-u.ac.jp (Y.Oki); Reiko1122@hiroshima-u.ac.jp (R.K.); abey@hiroshima-u.ac.jp (Y.A.); tsuga@hiroshima-u.ac.jp (K.T.)

**Keywords:** surface topography, titanium surface modification, o-phospho-l-threonine

## Abstract

Implant surface topography is a key factor in achieving osseointegration. l-Threonine can be chemically and stably bonded to titanium surfaces by phosphorylation. This study investigated the degree of in vivo osseointegration of an implant with a novel o-phospho-l-threonine (p-Thr)-binding surface. MC3T3-E1 cells were seeded on the p-Thr binding surface and machined surface disks, and initial cell attachment was evaluated. p-Thr-binding and machined surface implants were tested in vivo by implantation into the femurs of three male New Zealand white rabbits, and the osseointegration was assessed by measurement of removal torque (RT) and bone-implant contact (BIC) ratio. Initial cell attachment was greater for the p-Thr-binding than for the machined surface implant group (*P* < 0.05). In addition, RT and BIC values were higher for the p-Thr-binding surface than for the machined surface (*P* < 0.05). These results indicate that our implant with a p-Thr-binding surface can achieve enhanced osseointegration.

## 1. Introduction

The clinical success of oral implants is related to their early osseointegration, which in turn depends on different factors, including implant surface topography and design [1]. Surface topography plays a role in the osteointegration of implants [2]. The most common topographical modification is surface roughness, which is achieved by acid etching, sandblasting, or oxidization. The microtopography provides accelerated differentiation of osteogenic cell [3]. The roughness of dental implant surfaces (also known as the *S_a_* value) can be classified as smooth (0.0–0.4 µm), minimally rough (0.5–1.0 µm), moderately rough (1.0–2.0 µm), or rough (>2.0 µm) [4]. Many studies have reported that a rough surface promotes bone anchoring and biomechanical stability of implants [5] and enhances cell initial attachment and differentiation into osteoblasts [6,7]. These modification methods affect osteogenic cells activity because they modify the adsorption of proteins from biological fluids. Positively charged serum proteins attach to negatively charged titanium surfaces because of a difference in electrostatic potential [8]. Osteoblasts are then adsorbed onto the titanium, with integrin expressed by osteoblasts acting as receptors for the serum protein, thereby initiating bone formation. Thus, the binding of protein to the implant surface can enhance osseointegration. Rough surfaces are also superior to smooth surfaces in terms of osseointegration. In our previous study, we showed that stability was lower for implants with a machined surface than for those with a TiUnite^TM^ (Gothenburg, Sweden) surface (*S_a_*: 0.9 vs. 1.1 µm) [9]. However, greater surface roughness increases biofilm formation on the implant surface and abutment, thereby increasing the risk of peri-implantitis [10,11]. There is therefore a need for methods that enhance bone formation while increasing resistance to peri-implantitis. In our previous study, a phosphorylated amino acid (o-phospho-l-threonine (p-Thr)) was bonded to a titanium surface treated with HCl [12]. To take advantage of this material, in the present study, we designed a novel type of implant surface that promotes cell attachment and has a smoother surface topography. We speculated that p-Thr on the implant surface would enhance cell attachment and osteoblast activity in vivo, and tested this hypothesis by evaluating the osseointegration of the implants in rabbits. Therefore, the purpose of the present study was to investigate the effect of a novel p-Thr binding surface on osseointegration in vivo.

## 2. Results and Discussion

### 2.1. Results

#### 2.1.1. Evaluation of Surface Roughness

**Figure 1:**
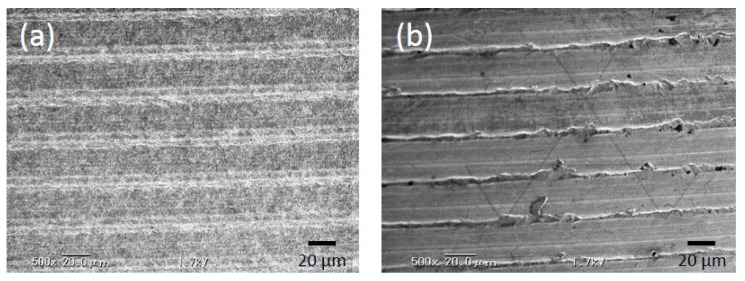
Scanning electron microscopy (SEM) analysis. (**a**) p-Thr-binding surface; (**b**) Machined surface. Both groups showed traces of machine tuning. The p-Thr-binding surface had a finer surface structure than the control.

p-Thr-binding and machined (control) surfaces were examined by scanning electron microscopy (SEM) (Figure 1).

Both groups showed evidence of machine turning, but the p-Thr binding surface was rougher than the control surface (*R_a_*: 0.41 ± 0.01 vs. 0.24 ± 0.02; *P* < 0.001) (Table 1).

#### 2.1.2. Measurement of Initial Cell Attachment

**Figure 2:**
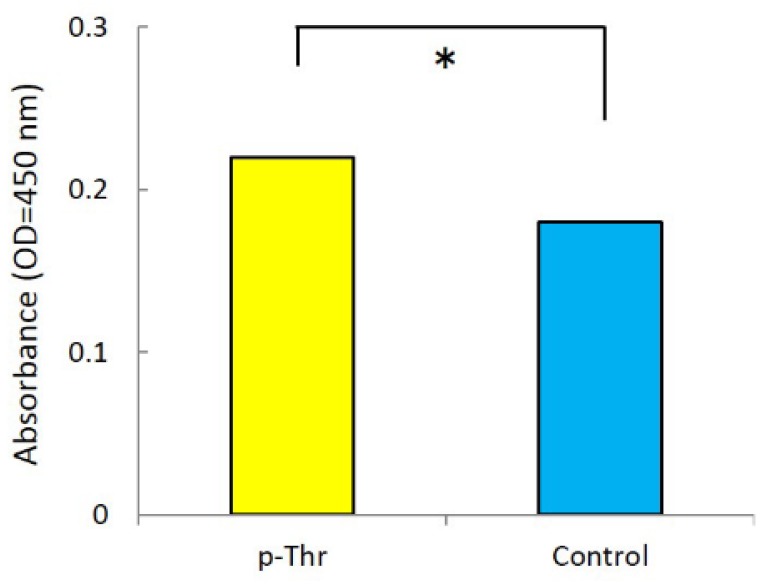
Initial cell attachment values. The p-Thr-binding group showed higher cell attachment than the control (*P* < 0.001).

The degree of initial cell attachment to the p-Thr-binding surface and machined surface was evaluated after 24 h of incubation (Figure 2). Cell attachment was greater in the p-Thr binding surface than in the control (0.22 ± 0.00 vs. 0.18 ± 0.00; *P* < 0.001).

#### 2.1.3. Measurement of Removal Torque and Bone-Implant Contact

**Figure 3:**
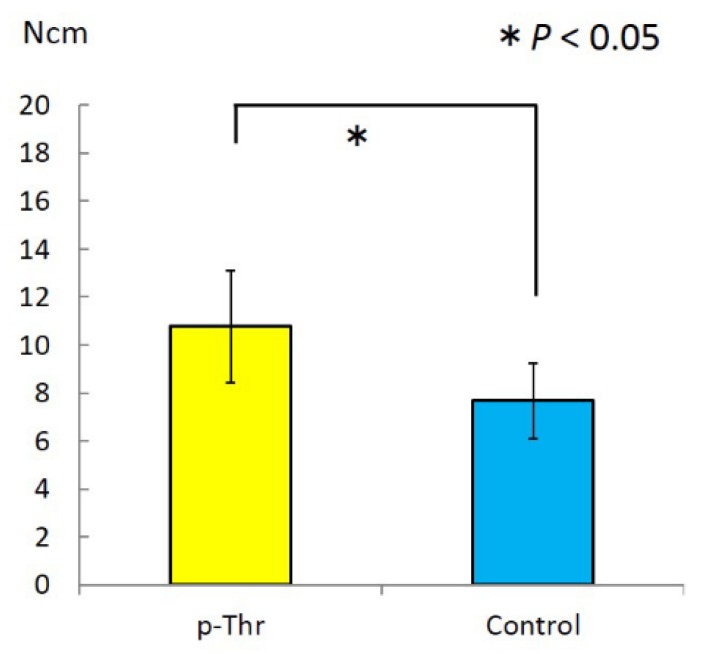
Removal torque (RT) values. The p-Thr-binding group showed a higher RT value than the control (*P* = 0.011).

Removal torque (RT) was measured using a digital torque gauge (Figure 3). The RT value was higher in the p-Thr-binding condition than in the control (10.77 ± 2.34 vs. 7.67 ± 1.59 N·cm; *P* = 0.011).

**Figure 4:**
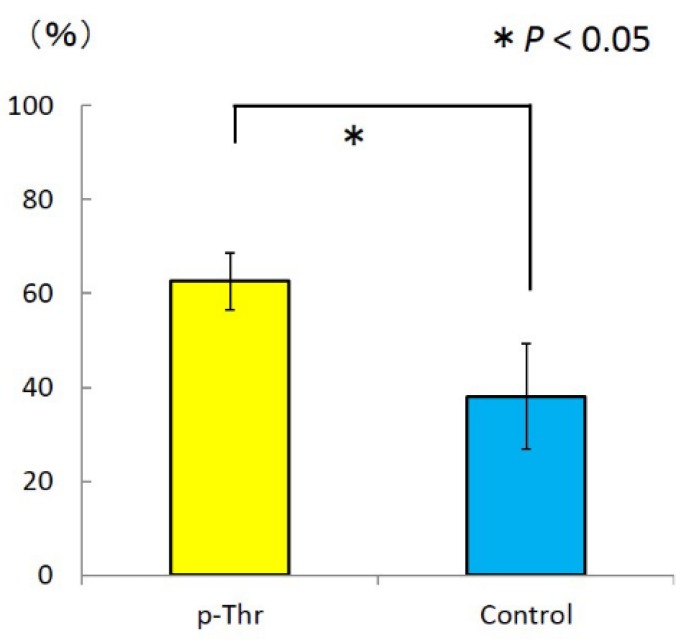
Bone-implant contact (BIC) ratio. The p-Thr-binding group showed a higher BIC ratio value than the control (*P* = 0.016).

A similar trend was observed for the bone-implant contact (BIC) ratio (62.2% ± 6.1% vs. 38.1% ± 11.3; *P* = 0.016) (Figure 4).

#### 2.1.4. Histological Observations

**Figure 5:**
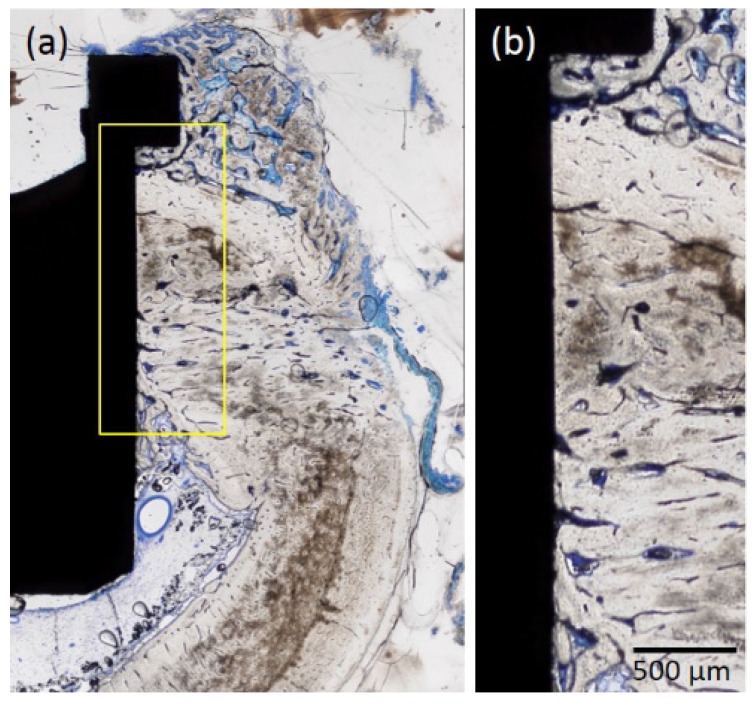
Histological analysis of specimens of the p-Thr-binding implant. (**a**) Osseointegration was observed at the bone/implant surface interface. Bone formation occurred towards the cortical bone portion near the bottom of the implant, where the bone marrow was located; (**b**) The implant surface made contact with bone at the collar portion. Original magnification: 40×; Toluidine Blue staining.

**Figure 6:**
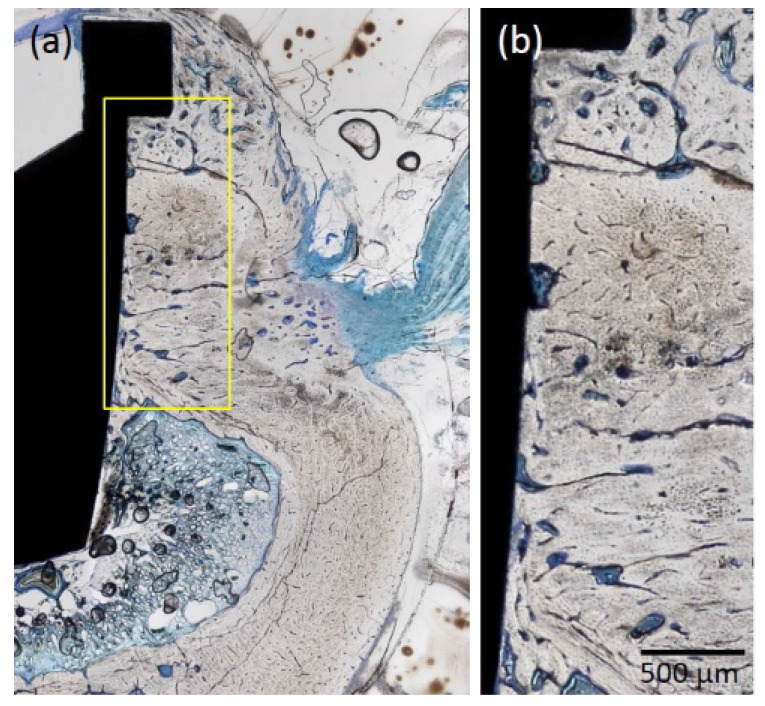
Histological analysis of specimens from the machined implant (control). (**a**) Osseointegration was observed at the bone/implant surface interface; (**b**) The implant surface made contact with bone, but to a lesser degree than that observed for the p-Thr-binding surface. Original magnification: 40×; Toluidine Blue staining.

Osseointegration was detected for both types of implant (Figure 5 and Figure 6). The p-Thr binding surface made contact with bone to a greater degree than the control implant, particularly at the collar portion of the implant surface; bone formation occurred towards the cortical bone portion near the bottom of the implant, where the bone marrow was located.

### 2.2. Discussion

The results of this study indicate that a p-Thr-binding surface can increase osseointegration as determined by RT and BIC values. Rough implant surfaces can achieve greater primary stability because of increased contact between the surface and surrounding bone, which enhances osseointegration by stimulating bone growth, thereby reducing the risk of implant failure during the early healing phase [13]. Recent studies have suggested that rough surfaces are associated with a higher risk of peri-implantitis than smooth surfaces over a long period, as they also permit attachment of bacterial cells [14,15]. However, smooth and rough surfaces are similar in terms of osseointegration capacity at secondary stability during the healing phase [13]. The extent of bone resorption resulting from experimental peri-implantitis was greater for implants with a TiUnite surface (*R_a_* 1.1) than for those with a machine-tuned surface [14].

Phosphate has been found to have a high affinity for titanium oxide surfaces [16]. Phosphoric acid molecules covalently attached to a titanium surface may form a scaffold for new bone formation, leading to bonding between implant and host tissue [17]. Amino acids such as l-serine, l-tyrosine, and l-threonine are low-molecular weight molecules that can be bonded to a titanium surface by phosphorylation. In particular, the peptide bond in l-threonine is rarely hydrolyzed under physiological conditions. Our previous study demonstrated that p-Thr could be chemically bonded to a titanium surface treated with HCl. However, the surface topography was slightly rough because of the HCl treatment. In the present study, Ra of the p-Thr binding implant was 0.41; however, the degree of roughness was classified as smooth [4]. In contrast to the untreated control surface, the p-Thr-binding surface promoted cell attachment in the healing phase, which induced bone formation on the surface. Osseointegration was detected in both groups; however, BIC was greater in the p-Thr binding group. This indicates that the osteoinduction capacity of the p-Thr binding surface was superior to that of the machined surface.

Implant stability reflects osseointegration and is considered an accurate measure of the success of an implant [18]. Osseointegration can be evaluated by resonance frequency analysis (RFA), a noninvasive method that continuously measures implant stability to determine whether a sufficient amount of bone surrounds the implant and whether the two materials have achieved integration [19,20]. However, this method has limited applications, for instance in commercial dental implants. Furthermore, RFA is conducted on a limited number of cross-sections and may not reflect the integration of the whole implant. To evaluate osseointegration in our animal model, we relied on histological observation and histomorphometric measurements. Specifically, we measured BIC and RT and found that the values for both parameters were higher for the implant with the p-Thr-binding surface than for the control, indicating that more bone was formed on the former surface. BIC and RFA values are related, and a positive correlation exists between increases in the implant stability quotient and BIC [21,22]. RT also provides a quantitative assessment of osseointegration of the entire implant, and is influenced by cortical bone thickness [23]. Moreover, surface topography (including roughness) influences cell attachment and differentiation and can modulate the speed of bone formation around the implant during the healing phase, explaining the higher BIC and RT values for the p-Thr-binding surface than for machined implants.

Based on the results presented here, we suggest that osseointegration can be effectively achieved with the p-Thr-binding surface implant in vivo. Future studies will evaluate the resistance of implants with rough surfaces to peri-implantitis to clarify the advantages of this novel type of surface.

## 3. Materials and Methods

### 3.1. Fabrication of p-Thr-Binding Surface

**Figure 7:**
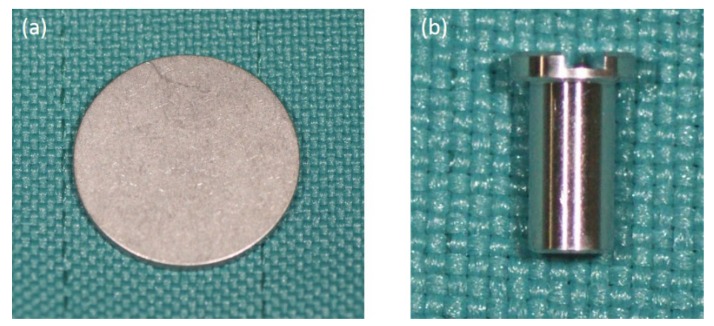
Samples of pure machined titanium. (**a**) Disk type: diameter = 13.0 mm; height = 1.0 mm; (**b**) Implant type: diameter = 3.0 mm, length = 5.0 mm.

The p-Thr binding surface was fabricated as described in a previous study [12]. Machined surface titanium disks (diameter: 13.0 mm, height: 1.0 mm) and the implant body (diameter: 3.0 mm, length: 5.0 mm) were custom-fabricated (Figure 7) from pure titanium (JIS 2 grade; Nishimura Co., Fukui, Japan), and then subjected to ultrasonic cleaning with 10 N HCl for 30 min, followed by rinsing with ultrapure water. The disks and implant were immersed in 50 mM p-Thr (molecular weight: 181.08; Sigma-Aldrich, Tokyo, Japan) at 37 °C for 12 h, then cleaned again with ultrapure water. The untreated disks and implants were used as a control.

### 3.2. Scanning Electron Microscopy and Surface Roughness Measurement

The *R_a_* of each sample was evaluated by confocal laser scanning microscopy (VK-8500; Keyence, Osaka, Japan). The *R_a_* value (µm) was defined as the average value of five different areas (100 × 100 µm^2^).

### 3.3. Initial Cell Attachment

MC3T3-E1 osteoblast-like cells were seeded on the p-Thr binding surface and machined surface titanium disk at a density of 1.0 × 10^5^ cells/well and cultured in Dulbecco’s Modified Eagle Medium containing 10% fetal bovine serum and 1% penicillin-streptomycin for approximately 24 h at 37 °C under a 5% CO_2_ atmosphere to allow initial attachment. Cell Counting Kit-8 (Dojindo Laboratories, Kumamoto, Japan) was used to measure the absorbance of the p-Thr binding surface at a wavelength of 450 nm.

### 3.4. In Vivo Assessment of Osseointegration

The experimental protocol conformed to the regulations established in the current version of the Japan Law on the Protection of Animals. The study was approved by the Research Facilities Committee for Laboratory Animal Science at Hiroshima University School of Medicine, Hiroshima, Japan (A-11-5-3). Three male New Zealand White rabbits (17 weeks old, 3.0–3.5 kg) were used. Surgeries were performed under general anesthesia with sodium pentobarbital (Somnopentyl, 10 mg/kg by intravenous injection; Kyoritsu Seiyaku Corp., Tokyo, Japan).

**Figure 8:**
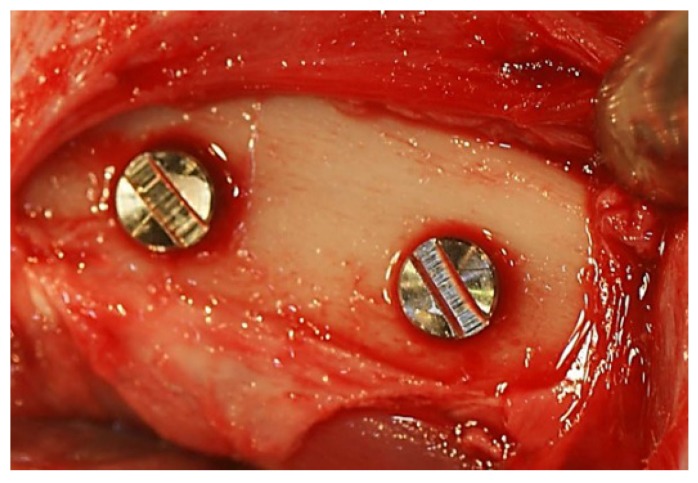
Implant placement. Implants were inserted into the bone socket in the bilateral femurs.

Muscle and periosteal flaps were made on the left and right femurs, and two implant sockets (diameter: 3 mm, depth: 5 mm) were prepared on each side. The p-Thr binding surface and machined implants were placed in the right and left bone sockets, respectively (*n* = 6) (Figure 8), and 4 weeks later, the RT of each implant was recorded using a digital torque gauge (BTG-E100CN; Tonichi, Tokyo, Japan) (*n* = 3).

The animals were sacrificed after measurements were obtained, and bone tissue blocks including the implant were harvested and immediately fixed in 10% buffered formalin for 10 days. Tissue blocks containing the implant were dehydrated in a graded series of ethanol, cleared with a styrene monomer, and then embedded in light-polymerized polyester resin (Technovit 7200 VLC, Kulzer, Hanau, Germany). The resin block was photopolymerized (BS5000, Exakt Aparatebau, Norderstedt, Germany), and the specimens were cross-sectioned with a high-precision diamond disk to obtain sections with a thickness of 200 µm. Non-decalcified specimens were ground to a thickness of approximately 70 µm (MG5000, Exakt Aparatebau), and sections were stained with Toluidine Blue.

### 3.5. Histological and Histomorphometric Evaluation

Sections were visualized by light microscopy (BZ-9000; Keyence, Osaka, Japan). Images were digitized, and histomorphometric analysis was performed using ImageJ software (National Institutes of Health, Bethesda, MD, USA). The BIC ratio was determined using ImageJ as the ratio of the contact length of the newly formed bone relative to the total length from the bottom to the top of the implant (*n* = 3).

### 3.6. Statistical Analysis

Data are presented as the mean ± standard deviation and were analyzed using Mann–Whitney *U* test. Statistical significance was defined as *P* < 0.05.

## 4. Conclusions

These results indicate that our implant with a p-Thr-binding surface can achieve enhanced osseointegration.

## Figures and Tables

**Table 1:** Surface roughness.

Group	*R_a_* µm (SD)
p-Thr	0.41 (0.01) *
Control	0.24 (0.02)

SD: standard deviation; * *P* < 0.001.
